# Leadership for Public Health 3.0: A Preliminary Assessment of Competencies for Local Health Department Leaders

**DOI:** 10.3389/fpubh.2017.00272

**Published:** 2017-10-16

**Authors:** Emmanuel D. Jadhav, James W. Holsinger, Billie W. Anderson, Nicholas Homant

**Affiliations:** ^1^College of Health Professions, Ferris State University, Big Rapids, MI, United States; ^2^College of Public Health, University of Kentucky, Lexington, KY, United States; ^3^College of Pharmacy, Ferris State University, Big Rapids, MI, United States

**Keywords:** public health leadership, public health management, leadership workforce development, leadership training, public health education

## Abstract

**Background:**

The foundational public health services model V1.0, developed in response to the Institute of Medicine report *For the Public’s Health: Investing in a Healthier Future* identified important capabilities for leading local health departments (LHDs). The recommended capabilities include the organizational competencies of leadership and governance, which are described as consensus building among internal and external stakeholders. Leadership through consensus building is the main characteristic of *Democratic Leadership*. This style of leadership works best within the context of a competent team. Not much is known about the competency structure of LHD leadership teams. The objectives of this study characterize the competency structure of leadership teams in LHDs and identify the relevance of existing competencies for the practice of leadership in public health.

**Materials and methods:**

The study used a cross-sectional study design. Utilizing the workforce taxonomy six management and leadership occupation titles were used as job categories. The competencies were selected from the leadership and management domain of public health competencies for the Tier -3, leadership level. Study participants were asked to rank on a Likert scale of 1–10 the relevance of each competency to their current job category, with a rank of 1 being least important and a rank of 10 being most important. The instrument was administered in person.

**Data:**

Data were collected in 2016 from 50 public health professionals serving in leadership and management positions in a convenience sample of three LHDS.

**Results:**

The competency of most relevance to the highest executive function category was that of “interaction with interrelated systems.” For sub-agency level officers the competency of most relevance was “advocating for the role of public health.” The competency of most relevance to Program Directors/Managers or Administrators was “ensuring continuous quality improvement.” The variation between competencies by job category suggests there are distinct underlying relationships between the competencies by job category.

## Introduction

Public Health 3.0 recommends that local health department (LHD) leaders use leadership and governance competencies that allow them to broadly impact community health outcomes (1–3). The foundational public health services model V1.0, developed in response to the IOM report *For the Public’s Health: Investing in a Healthier Future* (4), identified consensus building among internal and external stakeholders as one of the foundational organizational competencies of leadership and governance. Leadership through consensus building is the hallmark of Democratic Leadership and works best in the context of a competent leadership team, i.e., a competency structure of diverse competencies that enables the leader to lead by building consensus (5–7). In LHDs, not much is known about the nature or competency structure of their leadership teams. The objective of this study was to characterize the competency structure of leadership teams in LHDs and identify the relevance of existing competencies to the practice of leadership in public health.

The importance of characterizing the competency structure of the leadership team in LHDs is integral to developing training and educational materials that advance the knowledge and skill requirements of the current and future public health workforce, especially since the quality and preparedness of the public health workforce is dependent on the relevance and quality of its training and education (8). In addition to training and education, workforce quality is strongly related to experience that includes work functions and on the job training. Most public health workers who are inadequately trained for their jobs tend to adapt and learn on the job. The 2011–2012 Society for Human Resource Management report on human capital benchmarking determined that the total cost of replacement including training and loss of productivity ranges from 90 to 200% of the replaced employee’s annual salary (9), suggesting that the cost of on the job training is as important, if not more so, than the cost of training a replacement.

Several reports (10–12) on the education of public health professionals have underscored the need for competency-based management expertise in the practice of public health, implying that the public health workforce would benefit from competency informed job descriptions that reflect competency-based training and education (13). The result was the development of the 2015 Public Health Workforce Taxonomy (PHWT) (14) and 2014 Core Competencies for Public Health Professionals (CCPHP) (15). There is, however, a gap in knowledge between the relevance of the suggested competencies and the various leadership job categories. Findings from our study will provide information for future efforts directed toward the development of leadership capacity in LHDs.

### Background

In the practice of public health, workplace success is defined as the delivery of health-care services across different population levels (16). Empirical studies have identified that workplace success for public health agencies require competently trained leaders (11, 17) who can guide and facilitate the delivery of health-care services. Recent advances (15, 18) in the discipline of leadership and management in public health practice present a unique opportunity for developing training and educational materials to facilitate training leaders for workplace success (19).

In 2000, through the public health leadership competency framework, the National Public Health Leadership Development Network (NLN) made one of the earliest efforts to focus interest on competent public health leadership. This initial effort of NLN identified 79 leadership competencies (20) through the use of sequential workgroup assignments to network members of the NLN. This was followed by the accreditation initiative of the Public Health Accreditation Board (PHAB), which developed accreditation standards for public health agencies. PHAB released its first set of official guidelines in 2007, which included an emphasis on the development of a competent workforce (20). To intentionally integrate the changes in the public health environment, in 2013 PHAB released version 1.5 (21), which places an intentional emphasis on leadership development. To guide the workforce development requirements of accreditation the National Council on Linkages (NCL) in 2014 released version 2.0 of its competencies for public health professionals. The NCL report categorized the competencies by Domains and Tiers to represent different career stages for public health practitioners (15), defining three Tiers: entry level staff (Tier 1), supervisory level staff (Tier 2), and executive level staff (Tier 3). The leadership team is categorized by Tier III of the LHD workforce. In 2014, the PHWT was developed as a tool to systematically categorize workforce characteristics by job category (18). It was tested for reliability by the 2015 Public Health Workforce Interests and Needs Survey (14).

The recent development of knowledge resources for competency-based leadership in LHDs is a reflection of the overwhelming lack of competency-based instruction (13), which has not produced trained competent public health leaders (22). The findings from this study will inform the intentional effort (23) that has been set in motion to promote an evidence-based approach to ensuring a competent workforce. There is little doubt that the lack of competent leadership is of concern to the practice of public health, even more so the nature of its leadership, since the largest proportion of the public health workforce is composed of administrative personnel (24) that perform general management operations, making the proposed study pertinent for meeting the training and practice needs of those on the frontline of public health leadership.

## Materials and Methods

During 2016, in this cross-sectional study, 50 participants in leadership and management positions from three Michigan LHDs responded to the survey instrument. To approximate for variation in agency workforce capacity and infrastructure, a purposive sample of three Michigan LHDs (small, medium, and large) were recruited. The self-selection random sampling method was utilized to recruit participants in the study. The executive leadership of each of the three LHDs publicized the need for study participants. The criteria for participation required that the participants must be part of the executive leadership team; i.e., senior leaders engaged in defined management functions. Paper surveys were provided directly to each participant. The principal investigator and a research assistant administered the surveys. No incentives were provided to the participants. The anonymity of the survey participants was maintained since no identifying information is included in the survey analysis.

### Instrument

The instrument consisted of seven questions. Participants were asked the job title of their previous position, the number of years they had been in their current position, and their highest attained educational level. The participants also indicated that their current roles entail the supervision of others.

In order to characterize the structure of the competencies of the LHD leadership team and to identify their relevance, a survey cross-walk question was created. The question quantified ten leadership competencies by five different job categories within an LHD setting. The PHWT was utilized to develop the job categories which were pre-populated on the cross-walk question as Health Officer, Deputy Director, Department/Bureau Director, Program Director, and Public Health/Program Manager. The competencies used to cross-walk the job taxonomy were selected from the leadership and management domain of Tier 3 from the CCPHP. Participants were asked to rank on a Likert scale of 1–10 the relevance of each competency of their current job, with a rank of 1 being the least important and a rank of 10 being most important. To control for instrument measurement error, no rank could be used twice; e.g., rank 2 could not be used for more than one competency. Since there was only one Deputy Director, this job was combined into the Health Officer group.

Several open-ended discussions with the executive leaders within the three LHDs took place. These discussions identified that Tier 1 and Tier 2 employees in the LHDs were functioning in Tier 3 job categories. For example, these employees participate in Tier 3 workforce meetings and they coordinate activities, but they do not formally perform any management functions. To quantify this finding, a question identifying the distribution of management functions was included in the survey. This question was answered by the participants allocating a certain percentage of their job role to nine management functions. The discussions with the executive leaders identified nine management functions from those described by Gulick and Urwick (25) and the essential public health services (26).

### Data Analysis

The first level of analysis consisted of a review of the descriptive data collected. Percentages and sample sizes are reported for all questions not related to the competency framework. The second level of analysis focused on the competency framework question. To visualize the competency framework question, a stacked bar chart is used to display the average Likert scale for the aggregated job taxonomies for each competency and a second stacked bar chart displays the average Likert scales for each of the five jobs. The Likert scale responses to the competency framework question were averaged in order to determine how relevant each competency was to the participants. The most relevant competencies for each job category will be discussed in the Results and Discussion sections. The data and visual analysis were performed with SAS version 9.4 and Tableau 10.2.

## Results

### Leadership Team Characteristics

A total of 50 responses were received from the survey instrument. The summary of survey information is shown in Table 1. The distribution of participant characteristics displayed in Table 1 is similar to the profile of the national workforce in LHDs (27). Most participants were female (76%) and the highest degree obtained for most participants was a Masters’ degree (48%). An equal number of participants had tenure of five or more years and/or less than 5 years in their current LHD position. Since the participants were members of the executive team, or performing job functions similar to an individual on an executive team, it was not surprising that approximately 90% of all participants’ jobs involved some level of supervision. Most of the participants in the study group were Public Health/Program Managers (37%), followed by Program Coordinators/Administrators (19%), Program (17%) or Department Directors (17%), and Health Officers (10%).

**Table 1:** Leadership team characteristics.

Characteristics	Percent (*n*)
**Gender**	
Male	24 (12)
Female	76 (38)
**Highest Degree**	
Doctoral	4 (2)
Master’s	48 (24)
Baccalaureate	36 (18)
Associate’s	6 (3)
Missing/don’t know	6 (3)
**Tenure**	
Less than 5 years	48 (24)
More than 5 years	48 (24)
Job Involves Supervision	
Yes	90 (45)
No	10 (5)
**Current position**	
Health officer	10 (5)
Department/Bureau Director (sub-agency level)	17 (8)
Program Director	17 (8)
Public health/Program Manager	37 (18)
Program Coordinator/Administrator	19 (9)

The distribution of management functions reflects the inclusion of more Tier 2 personnel in the make-up of the executive leadership team. The most frequently performed management functions, as shown in Table 2, were managing daily operations (21%), providing overall direction (20%), and planning the use of resources (10%). The less frequently performed functions were evaluation (9%), coordination (9%), and analyses of resources (9%), followed by organizing (8%), directing the resources (8%), and developing policies within the guidelines set by the board (6%).

**Table 2:** Distribution of management functions.

Distribution of management functions	Average percent
Plan the use of resources	10
Organize the use of resources	8
Evaluate the use of resources	9
Coordinate the use of resources	9
Direct the use of resources	8
Analyze the use of resources	9
Develop policies within guidelines set by board	6
Provide overall direction	20
Manage daily operations	21

### Characteristics of Competency Structure

**Figure 1:**
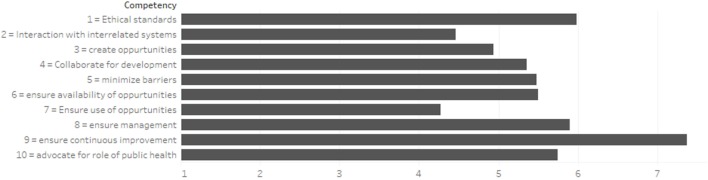
Characteristics of competency structure.

Figure 1 displays a stacked bar chart for importance of each of the reported relevant competencies for all the job categories. The lengths of the bars represent the average Likert scale for each competency. In aggregate, the most relevant competency was to “ensure continuous improvement.” The least relevant competency was to “ensure use of professional development opportunities.”

### Analysis of Competency Relevance

**Figure 2:**
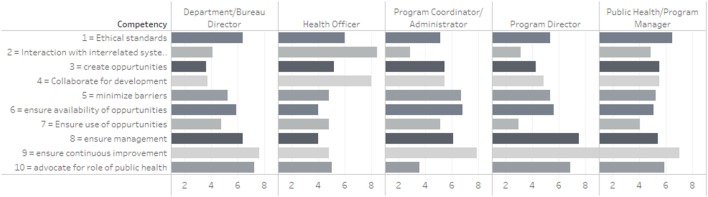
Analysis of relevant competencies by job category.

A pattern similar to that observed in Figure 1 emerges in the analysis of competency relevance by job category. Figure 2 displays a stacked bar chart representing the reported relevance of each of the competencies by job category. The “ensures continuous improvement” competency is the most important for every group except for Health Officers. The competency rated with the highest relevance to Health Officers was “interaction with interrelated systems.” For sub-agency level officers such as the Department/Bureau Directors, the competency of “advocating for the role of public health” ranked almost as high as “ensures continuous improvement.” The second most relevant competency after “ensures continuous improvement” was different for Program Directors, Managers, and Administrators. The second most relevant competency for Program Directors was to “ensure management of organizational change”; Public Health/Program Mangers rated “ethical standards” as the second most relevant competency; and Program Coordinator/Administrators valued “ensuring availability of professional development” as the second most relevant competency.

## Discussion

The analysis of the competency framework in this preliminary study suggests that within LHDs there is distinct variation in the relevance of leadership competencies based on job category. Members of the highest executive function category, Health Officers, appear to place more relevance on competencies that engage in inter-organizational activities. Sub-agency level job categories, such as Department/Bureau Directors, and Program Administrators/Mangers rate competencies that facilitate intra-organizational functions, such as operations and quality improvement as the more relevant. The findings corroborate other studies (28) that suggest competency-based job descriptions benefit job function. Developing job descriptions that focus on inter-organizational competencies for the highest executive function category results in enhanced interaction with other organizations and thus builds an environment that allows LHDs to work with relevant partners to achieve the Public Health 3.0 goal as Chief Health Strategist. The findings also suggest caution in expectations that are contrary to the level of job function; for example, expecting agency level managers to determine systemic functions may set up job frustration on the part of the leadership workforce. The study findings align with other studies suggesting that leadership failure may occur when there is a disconnect between the skills and competencies of the leaders and the requirements of the higher job responsibility (29). The study findings may be helpful in dealing with the challenge of aligning the public health education curriculum with the practice needs of leadership and management (17), as well as dealing with agency accreditation efforts.

While this preliminary study’s sample size limits generalization of the findings, it establishes the need for contemporary leadership development practices that are competency and evidence based. Another study limitation is the potential misclassification of job category specific competencies for the highest executive function category since the responses of the four self-identified LHD directors and one Deputy Director were combined in the study.

## Conclusion and Implications

In conclusion, the study findings indicate that there are distinct underlying relationships between the relevance of the various leadership competencies by job category. However, no clear distribution pattern characterizes the LHD leadership team, suggesting that existing LHD leadership teams lack a distinct competency structure. The lack of a distinct competency structure may hinder the ability of the leadership team to lead by consensus; an expectation of Public Health 3.0. Our findings reinforce the recommendation that specialized Public Health 3.0 training should be made available to LHD leadership teams, such that the development and description of job categories will be an effective workforce development exercise with the intentional effort of identifying competencies that align with job functions. While there are multiple factors that influence the performance of LHD leaders (30), a distinct, competency-based leadership team may assist in the success of its executive workforce members, and consequently improve the LHD’s executive retention effort. This study provides the foundation for additional studies to explore the performance of LHDs in which an executive team structure is present that is conducive to leading by consensus.

## Ethics Statement

This study protocol was approved by the “Ferris State University Institutional Review Board.” The study was exempt from documentation of written informed consent.

## Author Contributions

EJ conceived the study plan, analyzed data, and drafted and revised the manuscript. JH drafted and revised the manuscript. BA analyzed the data, wrote the statistical analysis, and revised the draft manuscript. NH drafted initial manuscript along with revising the draft manuscript. All authors reviewed and approved the final manuscript as submitted.

## Conflict of Interest Statement

BA serves as consultant/trainer for SAS. All other authors declare that the research was conducted in the absence of any commercial or financial relationships that could be construed as a potential conflict of interest.
